# Impact of the COVID-19 Pandemic on Pelvic and Acetabular Trauma: Experiences From a National Tertiary Referral Centre

**DOI:** 10.7759/cureus.15833

**Published:** 2021-06-22

**Authors:** Kunal Mohan, Patrick McCabe, Wafi Mohammed, Justin M Hintze, Hasnain Raza, Brendan O'Daly, Michael Leonard

**Affiliations:** 1 Department of Trauma & Orthopaedics, National Centre for Pelvic and Acetabular Surgery, Tallaght University Hospital, Dublin, IRL

**Keywords:** pelvis, fracture, covid-19, acetabulum, pandemic, trauma

## Abstract

Introduction

The coronavirus disease 2019 (COVID-19) pandemic has had a significant impact on daily life. Restrictions imposed to help minimise virus transmission have limited both population movement and employment, as well as altering the potential mechanisms of high-energy trauma. The objective of this study was to assess the impact of the COVID-19 pandemic on pelvic and acetabular trauma.

Materials and methods

A retrospective observational study of the incidence, causality, patient profile, fracture morphology, and treatment strategy of pelvic and acetabular trauma managed in a national tertiary referral specialist pelvic and acetabular centre between the 1^st^ of March and 1^st^ of August 2020 was undertaken and compared to corresponding time periods in the two preceding years.

Results

A total of 78 patients were referred for management following pelvic and acetabular trauma during the study period with a mean age of 52 years (SD +/- 24.2). Overall, 45% and 42% of patients were referred following isolated pelvic or acetabular fractures respectively. The most frequent mechanism of injury was a fall from height (>1m) (42%), with 53% of patients suffering from concomitant injuries and 32% requiring surgical management. While there was a statistically significant difference in mechanism of injury (P=0.026), there was no significant difference in overall incidence, fracture types, incidence of concomitant injuries, or overall proportion requiring surgical intervention during the study period when compared to previous years.

Conclusion

While some variation in the mechanisms of injury have been observed, the overall incidence, patient, fracture, and injury profiles associated with pelvic and acetabular trauma appear to have remained consistent during the COVID-19 pandemic. Additionally, the number and proportion of those requiring surgical treatment of these fractures have remained stable. Understanding the continued burden of these potentially severe injuries may help guide injury prevention, treatment, and resource allocation as the pandemic continues.

## Introduction

The novel severe acute respiratory syndrome coronavirus 2 (SARS-CoV-2) coronavirus disease 2019 (COVID-19) outbreak has resulted in one of the most profound healthcare challenges encountered in modern-day medicine [1]. Presumed to have originated in Wuhan, China, this respiratory disease was first reported in December 2019 and the subsequent widespread spread of cases worldwide resulted in the declaration of the COVID-19 outbreak as a global pandemic on the 11th of March 2020 [2].

Across the world, nationwide-specific approaches to both help limit the spread of COVID-19 within the community and to prevent both virus transmission as well as health systems from being overwhelmed by potentially increased demand created by the pandemic have been instigated [3]. In a societal context, these restrictions have significantly altered the manner in which people undertake their occupations, with many restricted solely to working from home at the height of restrictions [3,4]. Additionally, limitations on both international and national travel in attempts to curb transmission of COVID-19 have occurred to varying degrees and have impacted typical population mobility patterns [5]. Alterations to other aspects of life such as population gatherings, sporting, cultural, education and retail activities also occurred as part of the global response to COVID-19 [4], with the cumulative effect of all of these alterations being a definite impact on day to day population behaviour [6].

As a result of these changes, COVID-19 has had a tangible effect on current orthopaedic practice [7]. While re-prioritisation of elective orthopaedic at the height of the pandemic made up a part of initial systemic responses to COVID-19 [7], an alteration in both the volume and type of orthopaedic trauma encountered in daily practice was also predicted due to the anticipated alterations in typical population activity as a result of COVID-19 restrictions [8]. As the number of COVID-19 patients increased, management of orthopaedic trauma underwent reorganisation so to maximise both patient and staff safety and minimise the spread of the virus [9]. While orthopaedic trauma has continued to occur during the pandemic, an overall reduction in both the number of trauma patients, number of referrals to orthopaedic trauma services and number of orthopaedic admissions have been described in the current literature [10-12]. In addition, significant reductions in the overall number of orthopaedic operations undertaken have been identified [13]. An alteration in the typical mechanisms of orthopaedic trauma has also been witnessed, with reductions in the number of overall orthopaedic hospital admissions and referrals to the trauma emergency departments following both road traffic accidents (RTAs) and workplace incidents identified during the initial COVID-19 period [12,14]. Contrastingly, an increase in do-it-yourself (DIY) construction, falls from height and domestic-related injuries during COVID-19 restrictions have also been described [14,15].

While an overall alteration in orthopaedic trauma volume appears to have been observed during the peak of the COVID-19 pandemic [8,10,12,13], the trends for specific trauma subtypes appear to vary, with no statistically significant change in the operative incidence of periprosthetic fractures, soft tissue injuries, open fractures or spinal cord injuries identified in several studies [8,10,13]. Conversely, a statistically significant reduction in operative volume has been identified in lower limb fractures and simple fracture patterns [10], with differing results in regards to the incidence of both hip and upper limb fractures observed during the pandemic to date [8,10,12,13].

Pelvic and acetabular trauma constitutes between 3% and 8% of all fractures managed in the acute orthopaedic trauma setting, with an ever increasing incidence of these fractures encountered in modern-day practice [16]. Typically bimodal in age distribution [17], these fractures are associated with both significant morbidity and complications, particularly when requiring surgical treatment [18,19]. Due to the often complex nature of these fractures when occurring either in isolation or in the context of the high-energy polytraumatised patient, definitive management involving specialist treatment centres following initial stabilisation is recommended to help improve outcomes [20]. As a result, management of these fractures require significant resources and are thus associated with substantial direct and indirect costs to patients, the healthcare system and society alike [21].

While the burden of pelvic and acetabular trauma is potentially significant, there is to our knowledge a relative paucity of evidence to date evaluating the specific impact of the ongoing COVID-19 pandemic on pelvic and acetabular fractures at a national level. The objective of this study was, therefore, to quantify whether there has been any alteration in both the incidence of pelvic and acetabular fractures and the proportion requiring surgical treatment as a result of the pandemic, as well as to identify any potential effect that the pandemic has had on the patient, fracture and injury profiles typically associated with these types of trauma.

## Materials and methods

A retrospective, observational, single-centre study of patients sustaining pelvic and acetabular fractures was undertaken. This study was carried out in a national centre for pelvic and acetabular surgery, which serves as the solitary national tertiary referral specialist hospital centre for management of these injuries in the Republic of Ireland, receiving referrals nationwide from all hospitals managing acute orthopaedic trauma. Ethical approval was obtained from the hospital’s Research and Ethics Committee.

Patients referred for assessment for management of pelvic and acetabular fractures sustained between the 1^st^ of March 2020 and 1^st^ of August 2020 were identified through a prospectively maintained departmental referral database and deemed suitable for inclusion as the study group. Patients who suffered their index injuries outside of this time period were excluded from this study. This time period was selected to broadly correspond to the initial phases of the national response introduced following the start of the COVID-19 pandemic, with the first case of COVID-19 diagnosed nationally on the 29th of February 2020 [4]. Additionally, patients referred following pelvic and acetabular fractures sustained during the corresponding time period in both 2019 and 2018 were also identified through the same departmental referral database for inclusion as control groups for the purposes of comparison.

Between the 1st of March and 1st of August 2020, six distinct phases of national restrictions were instituted in response to the COVID-19 pandemic [4]. Each of these phases placed different limitations upon widespread aspects of society including but not limited to internal travel, economic activity and population gatherings and are broadly described in Table 1 [4]. Patients suitable for inclusion during the study period in 2020 were additionally subdivided by their temporal correspondence to specific restriction subphase.

**Table 1:** Generalised Summary of National Governmental 2020 COVID-19 Restrictions

Governmental Phase [4]	Dates	National Travel Activity	Economic Activity	Social Activity	Commercial/ Educational Activity
Containment Phase	1^st^-12^th^March	No restrictions.	No restrictions.	Self-isolation if symptomatic, contact tracing.	No restrictions.
Delay Phase	12^th^-27^th^March	No restrictions.	Reduction of workplace contacts, work from home if possible.	Gatherings ≤ 50 people indoor, ≤ 200 outdoor.	Closure of educational institutions.
Stay at Home Phase	27^th^March-18^th^ May	2km radius from home.	Essential work only otherwise work from home.	Gatherings outside of living unit prohibited.	Only essential retail open.
Easing of Restrictions: Phase 1	18^th^ May-8^th^ June	5km radius from home.	Return of outdoor work otherwise work from home.	Gatherings ≤ 4 people outdoors only.	Only essential & outdoor retail open.
Easing of Restrictions: Phase 2	8^th^ June-29^th^ June	Within county of residence or 20km radius from home .	Return to work if needed otherwise work from home.	Gatherings ≤ 4 people indoors, ≤ 15 outdoors.	All retail reopened.
Easing of Restrictions: Phase 3	29^th^ June-1^st^ August	No restrictions.	Return to work if needed otherwise work from home.	Gatherings ≤ 50 people indoors, ≤ 200 outdoors.	All retail reopened.

Demographic data recorded for those suitable for inclusion in either the study group (2020) or comparison groups (2019 and 2018) contained original referral hospital location, date of injury, gender and age. Patients underlying medical status was quantified using the American Society of Anaesthesiologists Physical Status Scale (ASA-PS). The primary study outcomes were extrapolated from the departmental referral database, medical notes and accompanying radiological investigations and are outlined in Table 2.

**Table 2:** Recorded Primary Data Outcomes

Primary Outcomes	
Total Number of Referrals	% of Total Referrals Requiring Surgery
Total Number of Referrals Requiring Surgery	% Presenting with Concomitant Injuries
Fracture Pattern: Isolated Pelvic Ring, Isolated Acetabulum, Combined Pelvis & Acetabulum, Isolated Ilium, Isolated Sacrum	Mechanism of Injury: Road Traffic Accident (RTA), High-Energy Fall from Height, Low-Energy Fall from Standing, Crush Injury, Atraumatic

The above information was recorded into an electronic database and divided into three distinct time periods (March-August 2020, March-August 2019 and March-August 2018 respectively). Those referred in 2020 were also subdivided by national restriction subphase.

Assessment of the recorded data included both qualitative and quantitative analysis. Descriptive statistics for qualitative analysis included number (n), percentage (%), mean and standard deviation (SD), allowing for both illustration of trends and comparison of the study group referred in 2020 to the two preceding control years as well as by national restriction subphase within 2020 itself. 

Quantitative analysis was undertaken using Statistical Package for the Social Sciences (SPSS) version 26 (IBM Corp, Armonk, NY) with a P value of <0.05 considered statistically significant. Comparison of the overall number of referrals, number of referrals undergoing surgical treatment and the proportion of those referred requiring surgical intervention was compared between the study group (2020) and two control groups (2019 and 2018) through a one-way analysis of variance (ANOVA). Evaluation of potential difference in the mechanism of injury, types of fractures and incidence of concomitant injuries in those referred between the study group (2020) and two control groups (2019 and 2018) was undertaken using cross-tabulation and chi-square analysis, with additional post-hoc analysis using Bonferroni correction to detect differences in column proportions in those outcomes displaying statistically significant differences.

## Results

Overall, 78 patients were referred for assessment for the management of their pelvic and acetabular fractures to the national centre during the study period of 1^st^ March to 1^st^ August 2020, with a corresponding rate of 0.51 pelvic and acetabular fracture referrals to the national centre per day. Of those included, 53 were males and 25 females at a male:female ratio of 68% to 32%, with an overall mean age of 52 years (SD +/- 24.2) (Table 3). Division of those referred in 2020 by subphase of national restrictions is further illustrated in Table 4. Overall ASA-PS grading of those included showed a predominance of either healthy patients or those with mild underlying systemic disease, with 85% (n=66) identified as either ASA-PS I or II (Table 5).

**Table 3:** Hospital Indicators and Patient Demographic Characteristics by Year

Characteristics	2020	2019	2018
Total Number of Pelvic & Acetabular Referrals	78	59	88
Number of Referrals/Day	0.51	0.39	0.58
Mean Age of Referrals (Years)	52	53	52
Male:Female Ratio	68:32	63:37	66:34
% of Referrals with Concomitant Injuries	53%	37%	53%
Number of Referrals Requiring Surgery	25	32	36
Number of Surgeries/Day	0.16	0.21	0.24
% of Total Referrals Requiring Surgery	32%	54%	41%

**Table 4:** Included 2020 Patient Demographics by Governmental Phase

	Total	Containment	Delay	Stay at Home	Easing of Restrictions 1	Easing of Restrictions 2	Easing of Restrictions 3
Total Number of Referrals	78	5	5	18	10	9	31
Number of Referrals/Day	0.51	0.45	0.33	0.35	0.48	0.43	0.94
Mean Age of Referrals (Years)	52	42	63	49	55	55	52
Male:Female Ratio	68:32	60:40	40:60	78:12	80:20	89:11	58:42
Number of Referrals Requiring Surgery	25	4	1	5	6	2	7
Number of Surgeries/Day	0.16	0.36	0.07	0.10	0.29	0.10	0.21
% of Total Referrals Requiring Surgery	32%	80%	20%	28%	60%	22%	23%

**Table 5:** % Included Patient ASA-PS Classification by Year ASA-PS: American Society of Anaesthesiologists Physical Status Scale.

	2020	2019	2018
ASA-PS I	59%	59%	51%
ASA-PS II	26%	29%	23%
ASA-PS III	10%	10%	26%
ASA-PS IV	5%	2%	0%

**Figure 1:**
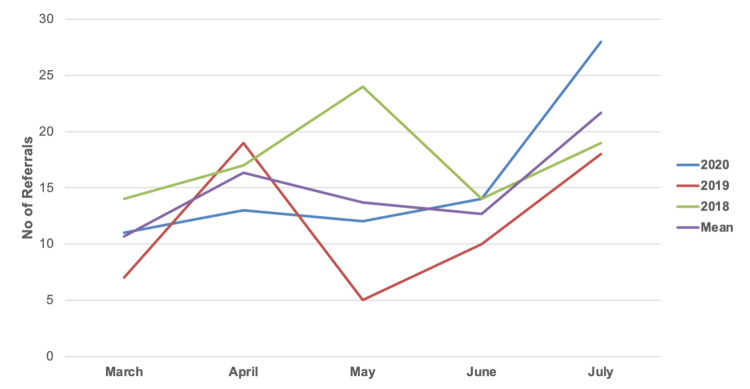
Total Number of Referred Patients by Month

Comparatively, 59 and 88 patients were referred for assessment for the management of pelvic and acetabular fractures between the 1st of March and 1^st^ of August in 2019 and 2018, respectively. One-way ANOVA analysis showed no statistical difference in the overall number of referrals in each year (P=0.204) (Table 6). The number of referrals per month across all three years is further illustrated in Figure 1.

**Table 6:** One-Way ANOVA Statistical Analysis of Hospital Indicators by Year ANOVA: analysis of variance.

	2020	2019	2018	P-Value
Total Number of Referrals	78	59	88	0.204
Total Number of Referrals undergoing Surgery	25	32	36	0.642
% of Total Referrals undergoing Surgery	32%	54%	41%	0.405

In 2019, the overall mean age of those referred was 53 (SD+/-22.8), with 63% (n=37) of these males and 37% (n=22) of these females, reflecting the profile of patients referred in 2020. Similarly, the mean age of those referred in 2018 was 52 (SD+/-22.0), with 66% (n=58) and 34% (n=30) of those suitable for inclusion male and female respectively, again resembling the profile of those referred in 2020. The underlying medical status of those referred in 2020 was also comparable to that witnessed in both 2019 and 2018, with 86% (n=52) and 74% (n=65) of patients either ASA-PS I or II in 2019 and 2018, respectively. Further descriptive comparison of referred patient profiles in between the study group and two control groups can be found in Table 3 and Table 5.

**Figure 2:**
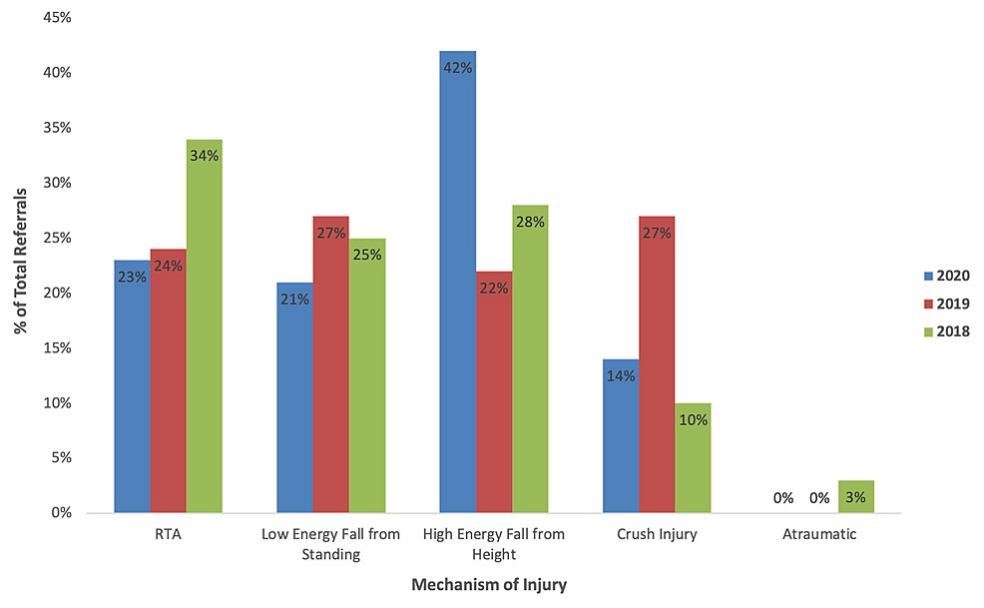
Mechanism of Injury by Year

In 2020, the most common causative mechanism of injury was following a high-energy fall from height, occurring in 42% (n=33) of patients. This was followed by RTAs (23%, n=18), low-energy falls from standing (21%, n=16) and crush injuries (14%, n=11). However, the most common mechanism of injury in 2019 was either a low-energy fall from standing or a crush injury (each 27%, n=16), with RTA (34%, n=30) the most common causative mechanism of injury in 2018. Overall illustration of mechanism of injury by % of total referrals per year is illustrated in Figure 2. Chi-squared analysis of column proportions showed a statistically significant difference in the proportion of mechanism of injury between each year (p=0.026), with additional post hoc analysis showing a statistically significant higher proportion in falls from height during the 2020 study period when compared to 2019 (p<0.05).

**Figure 3:**
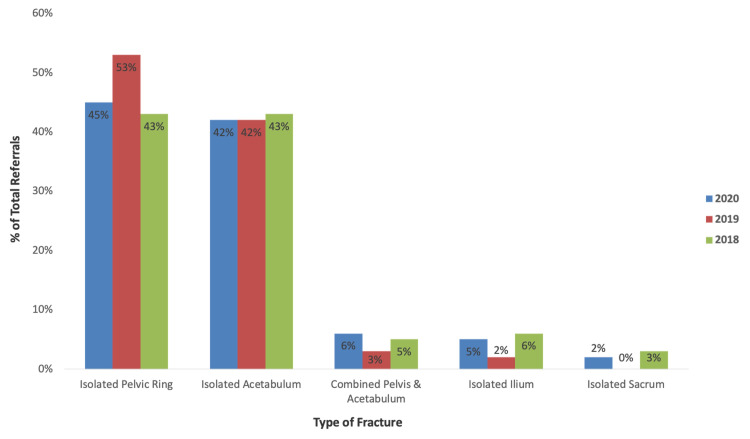
Type of Fracture by Year

Isolated fractures of the pelvic ring were the most predominant fracture type referred in 2020, occurring in 45% (n=35) of referrals. This was followed by isolated acetabular fractures, which occurred in 42% (n=33) of referrals. Combined pelvis and acetabular fractures, isolated ilium fractures and isolated sacrum fractures made up 6% (n=five), 5% (n=four) and 2% (n=one) of referrals encountered in 2020, respectively. Comparably, the most frequent fracture type encountered in 2019 were isolated pelvic ring fractures (53%, n=31) and isolated acetabular fractures (42%, n=25), with isolated pelvic ring and acetabular fractures each occurring in 43% (n=38) of referrals in 2018. A summary of the types of fractures encountered in each year is further illustrated in Figure 3. Chi-squared analysis of column proportions showed no statistically significant difference in the types of fractures encountered between each year (p=0.731).

Concomitant skeletal or visceral injuries to the referred pelvic and acetabular fractures were detected in 53% (n-41) of patients referred in 2020, with 37% (n=22) and 53% (n=47) of patients suffering from concomitant injuries in 2019 and 2018, respectively (Table 3). Overall, chi-squared analysis of column proportions showed no statistically significant difference in the proportion of patients sustaining concomitant injuries to their referred pelvic and acetabular fractures (p=0.115).

**Figure 4:**
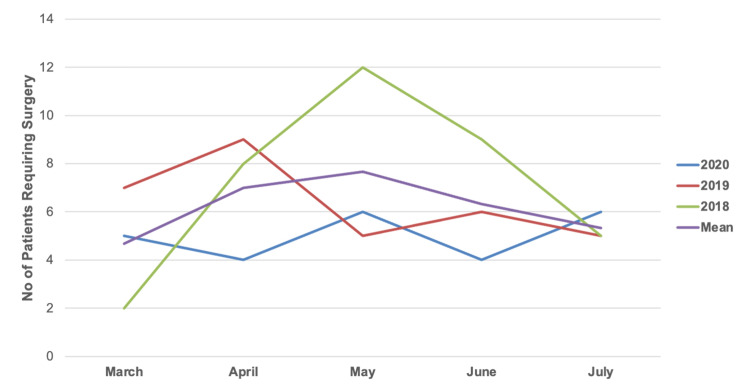
Total Number of Referred Patients Requiring Surgery by Month

Of the total number of patients referred following pelvic and acetabular fractures in 2020, 32% (n=25) required transfer and admission for surgical intervention following assessment. The overall surgical rate per day in 2020 was 0.16 (as seen in Table 3), with division of those operated upon in 2020 further divided by subphase of national restrictions in Table 4. In 2019 and 2018, 54% (n=32) and 41% (n=36) of total referrals required admission for surgical referral respectively. One-way ANOVA analysis showed no statistical difference in both the overall number of patients requiring surgical intervention (0.642) or the proportion of patients referred requiring surgery (p=0.405) between each year (Table 6). The number of patients requiring surgical intervention per month across all three years are further illustrated in Figure 4. 

## Discussion

COVID-19 has had an effect on the amount of trauma encountered in orthopaedic practice, with difference in the volume managed during COVID-19 when compared to previous years described in the literature [8,10,12,13]. Variations in the type of trauma exposure have also been reported, with increased rates of domestic-related injuries and decreased RTA and sports-related injuries described [14,22,23]. A combination of increased time restricted to home, seasonal weather changes, and an increase in DIY activities during COVID-19 lockdown has been described as being associated with a significant incidence in domestic-related traumatic events, with a particular prominence of falls from height such as ladders as a result of these behavioural changes [14,23].

A similar pattern appears to also occur in those suffering from pelvic and acetabular fractures, with the most important findings of this study identifying both a statistically significant difference in the types of mechanism of injury between each year and a statistically higher proportion of falls from height during the COVID-19 pandemic period when compared to the previous year, with falls from height the most frequent mechanism of injury precipitating in pelvic and acetabular fractures during the COVID-19 pandemic study period.

While there appears to have been an alteration in the mode of injury precipitating in pelvic and acetabular fractures during the pandemic identified in this study, there was no statistically significant difference in the overall incidence of patients referred for management of pelvic and acetabular fractures during the pandemic in this study when compared to prior years. Additionally, the encountered patient profile in regards to age, gender, and comorbid statuses seen in those sustaining pelvic and acetabular fractures during COVID-19 appeared comparable to both that witnessed in previous years as well as trends previously identified of those sustaining traumatic injuries nationally [24]. 

**Figure 5:**
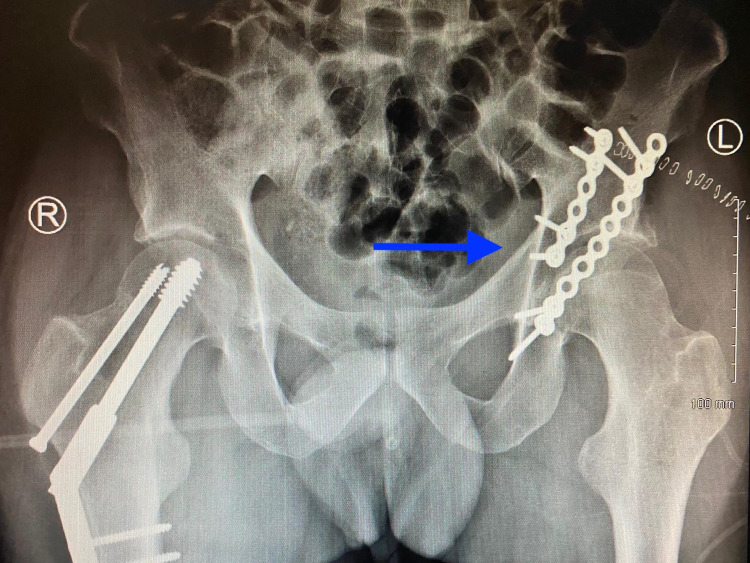
Plain Radiographic Example of a Left Acetabular Fracture Sustained During the COVID-19 Pandemic Requiring Surgical Fixation

Similarly, there appears to be no significant difference in the types of pelvic and acetabular fractures encountered during COVID-19 in this study, with the proportion of each fracture type appearing relatively consistent across each year. There was also comparable incidence of concomitant injuries in those referred for management of their pelvic and acetabular fractures, allowing for inference that while the type of traumatic insult may have altered during COVID-19, the degree and significance of injuries causing these types of fractures continue to persist, with the existence of other concomitant injuries being poorly prognostic for successful outcomes following these potentially severe fractures [25]. This hypothesis is further strengthened by the findings that during the COVID-19 study period, a comparable volume and proportion of all pelvic and acetabular fractures referred for assessment to the national centre in this study continued to require surgical treatment so as to best optimise outcomes (Figure 5).

As the COVID-19 pandemic continues, understanding of the resultant alterations in all facets of medicine will allow for stratification of the healthcare response and permit more effective management of further waves of the virus [26]. Pelvic and acetabular fractures are typically complex and significant injuries, often requiring substantial resources and specialist input so to maximise patient outcomes and minimise potential complications [18-20]. While there appears to have been an alteration in the mode of injury precipitating these fractures during the pandemic within the context of imposed societal restrictions, the overall proportion of pelvic and acetabular fractures managed appears to have remained consistent. Given the potentially significant sequelae of these injuries [18-20], understanding of the persistent burden of these injuries during this pandemic may help guide resource allocation and optimise trauma so as to insure optimal treatment of these injuries [14,27], particularly in regards maximising expedient and specialised care within the context of attempting to minimise virus transmission. 

Additionally, further understanding of the COVID-19 associated variations in precipitating injury patterns previously outlined in published orthopaedic literature [12,14,15,22,23] is of benefit in raising awareness and reducing the risk of further injury, particularly in regards to falls from height in the domestic setting [14]. While limitations to our study exist in regards to sample size, its retrospective design and potential for referral severity bias to the national centre for management of pelvic and acetabular fractures, we believe this study to be of importance as it allows reporting of the impact of the pandemic at a national level on these potentially severe injuries, thus possibly help guide future understanding of pelvic and acetabular fractures as the COVID-19 pandemic continues.

## Conclusions

While some variation in the causative mechanisms of injury has been observed, the overall incidence, patient, fracture, and injury profiles associated with pelvic and acetabular trauma appear to have remained consistent during the COVID-19 pandemic. Furthermore, the number and proportion of those requiring surgical treatment of these fractures have remained stable. Understanding the persistent burden of these potentially severe injuries during the global pandemic may help guide injury prevention, treatment, and resource allocation during the course of the pandemic, as well as help guide further epidemiological study of the variation in orthopaedic trauma encountered during this period as our understanding of the impact of COVID-19 continues to develop.
